# Stochastic Assembly of Bacteria in Microwell Arrays Reveals the Importance of Confinement in Community Development

**DOI:** 10.1371/journal.pone.0155080

**Published:** 2016-05-06

**Authors:** Ryan H. Hansen, Andrea C. Timm, Collin M. Timm, Amber N. Bible, Jennifer L. Morrell-Falvey, Dale A. Pelletier, Michael L. Simpson, Mitchel J. Doktycz, Scott T. Retterer

**Affiliations:** 1 Kansas State University, Manhattan, Kansas, United States of America; 2 The University of Tennessee, Knoxville, Tennessee, United States of America; 3 Oak Ridge National Laboratory, Oak Ridge, Tennessee, United States of America; Chengdu Institute of Biology, CHINA

## Abstract

The structure and function of microbial communities is deeply influenced by the physical and chemical architecture of the local microenvironment and the abundance of its community members. The complexity of this natural parameter space has made characterization of the key drivers of community development difficult. In order to facilitate these characterizations, we have developed a microwell platform designed to screen microbial growth and interactions across a wide variety of physical and initial conditions. Assembly of microbial communities into microwells was achieved using a novel biofabrication method that exploits well feature sizes for control of innoculum levels. Wells with incrementally smaller size features created populations with increasingly larger variations in inoculum levels. This allowed for reproducible growth measurement in large (20 μm diameter) wells, and screening for favorable growth conditions in small (5, 10 μm diameter) wells. We demonstrate the utility of this approach for screening and discovery using 5 μm wells to assemble *P*. *aeruginosa* colonies across a broad distribution of innoculum levels, and identify those conditions that promote the highest probability of survivial and growth under spatial confinement. Multi-member community assembly was also characterized to demonstrate the broad potential of this platform for studying the role of member abundance on microbial competition, mutualism and community succession.

## Introduction

Microbial communities impact our lives in dramatic ways. Taking on the role of both friend and foe, these communities shape our environment and ecosystems, fuel business and agriculture, and simultaneously support and perplex our healthcare system. Most communities are as dynamic as they are diverse, continuously adapting in their composition, organization and function to survive and thrive in changing environmental landscapes.

Within the community, microbes exchange materials, energy and information via metabolite transfer, diffusive chemical signaling (e.g. quorum sensing) and contact-mediated interactions (e.g. protein secretion) [1–3]. As a result, complex behaviors such as robustness (e.g. antibiotic resistance), efficient resource utilization, mutualism and enhanced biosynthetic capacity emerge. Community development is dramatically influenced by the chemical and physical landscape of the local environment [4]. Systems such as soil, packed bed reactors and medical device surfaces contain spatially-confined micro-environments or niches, where the path length for diffusive chemical signaling is significantly altered and where micro and nanoscale topology influence surface attachment events that impact community structure and function [5,6].

Conventional physiological assays of single species and multi-member cultures, carried out in large fluid volumes or over solid media, fail to address the impact of spatial organization and environmental heterogeneity on community development and function. Recent advancements in the capacity to characterize communities with improved spatial and temporal resolution have fueled a growing appreciation of the role that composition and physical architecture play on community dynamics. Advances in omics and sequencing technologies have been combined with the ability to image communities at the cellular and subcellular scales to provide a systems-level understanding of natural community function and adaptation [7]. Concurrently, a growing capacity to manipulate material and chemical environments across length scales, using micro and nanofabrication, has enabled studies that uncover the impact of hierarchical and heterogeneous structure on early-stages of community development and function [8]. For example, manipulation of surface architecture and spatial confinement at the length scale of single cells has been used to dramatically alter early colonization and self-regulated quorum signaling [9,10]. At the community level, studies into the role of confinement and microscale chemical heterogeneity has provided new insights into competition, cooperation, antibiotic resistance and succession [11–14].

Despite these advances, much is unknown about how microbial community members develop with respect to their neighbors and within their respective physical and chemical landscapes. This has inhibited our ability to assemble artificial communities of natural isolates or design synthetic communities of engineered organisms that exhibit the robustness and functional capacity of natural communities. These limitations are due, in large part, to our inability to systematically screen the vast parameter space of factors that influence ecological succession and function [15]. Member abundance, confinement, niche connectivity, and nutrient exchange are just a few of the factors that can influence such processes [16]. Discovery necessitates the development of screening tools that can rapidly and systematically explore this space in order to identify those conditions and combinations of community members that promote or inhibit heterogeneous growth, coordinate function and drive emergent behavior.

Toward these goals, several platforms have been developed that utilize micro- and nano-fabrication techniques to enable the study of uncultivable bacterial species, the study of competition or synergy in small bacterial communities [17], and studies of the effects of spatial structure and chemical environment on colonization [11,18]. Several approaches have been taken that confine small numbers or single microbial cells in small volumes, enabling these individual microbes or small colonies to be observed microscopically. Microfluidic droplet generators that encapsulate bacteria are a common approach [19–24]. Also used are arrays of microwells [25], like the million-well growth chip designed by Ingham et al. that was applied for use as a high-throughput screening tool [26]. Another interesting approach utilizes a ‘cell docking’ method that delivers microbes to arrays of different diameter wells, using microfluidic channels, potentially allowing different aspects of confinement to be analysed [25].

**Fig 1:**
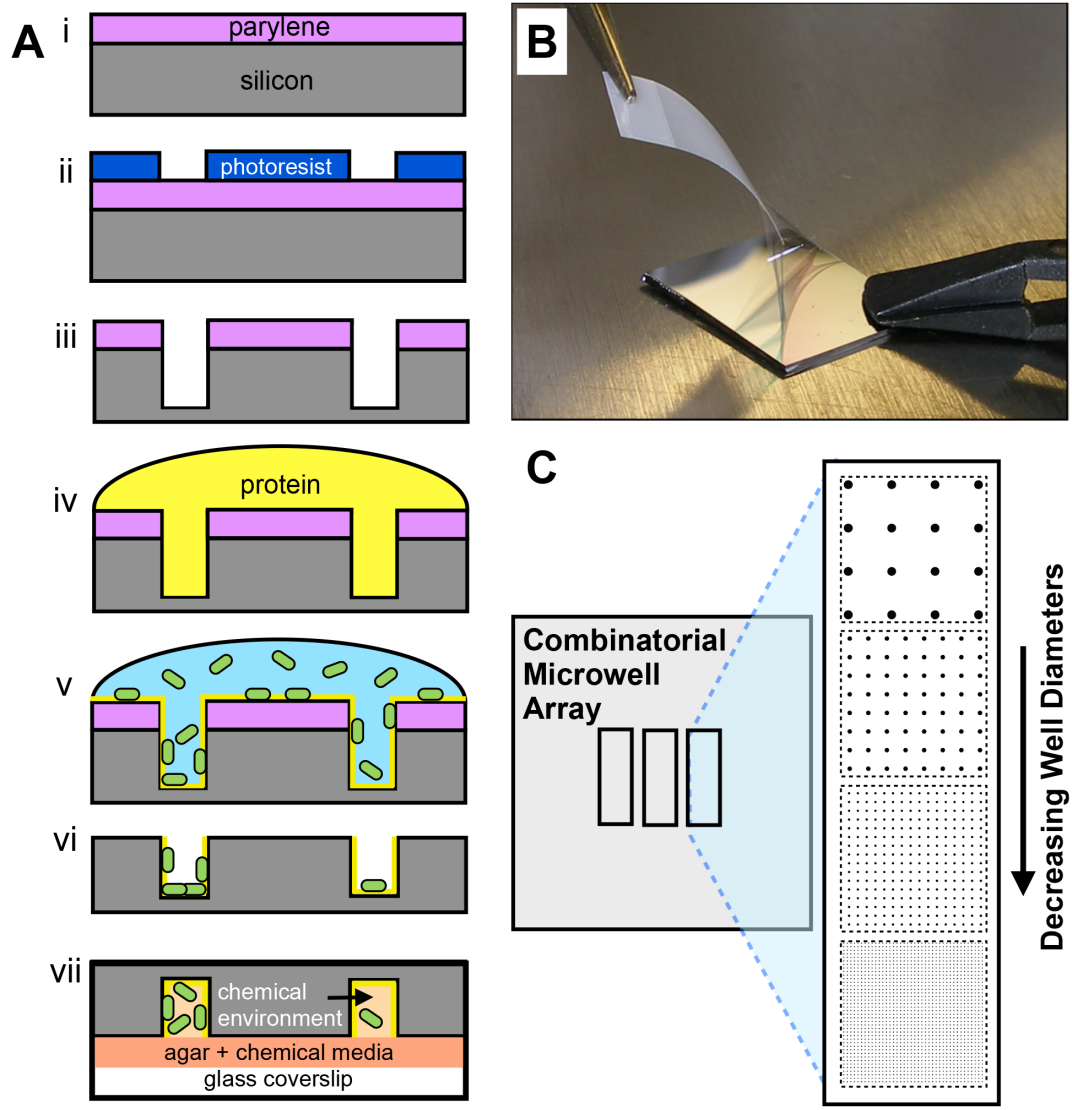
Microwell array fabrication and design. (A) Microwell fabrication process: (i,ii) Positive photoresist is patterned over parylene-coated silicon wafers using conventional photolithography. (iii) Dry etching is then used to etch parylene and then silicon to the desired well depth. (iv) The well surface is then modified with a protein layer then (v) a solution of bacterial cells. (vi) Parylene is removed from the substrate and (vii) the substrate is contacted with agar-coated coverslips loaded with the desired chemical media. (B) Dry lift-off procedure involving peel-off of the parylene mask (step vi). (C) Layout of a combinatorial microwell array substrate.

Here, we detail the development of a discovery platform that combines the high-throughput nature of the droplet technologies and the million-well growth chip mentioned above [26] with the flexibility in size and environmental control afforded by microfabricated wells described by Park et al. [25] This multi-diameter microwell array is designed to screen unique microbial communities for growth across a large range of population and environmental parameters. We describe a microfabrication approach that uses a polymer dry lift-off method [27–29] to assemble microbial populations into silicon microwells with controlled surface chemistry and physical features (Fig 1*A* and 1*B*). By varying the length scale of the well diameter (Fig 1*C*), we show that homogenous populations assemble into large (10^1^–10^2^ μm diameter) wells, while highly heterogeneous populations assemble into small (10^0^–10^1^ μm diameter) wells. Leveraging the high initial population dispersion driven by assembly into small wells, we screen unique *Pseudomonas aeruginosa* colonies to identify conditions that are either conducive or inhibitive to growth in spatially-confined environments. Finally, seeding with a binary system was characterized to demonstrate the utility of this platform for pairing interacting cells together in a controlled or randomized fashion. These results demonstrate a new, high-throughput methodology for screening population and environmental parameters for microbial growth.

## Materials and Methods

### Design and fabrication of microwell arrays

Two different microwell array layouts were used in these studies. The first layout contained six separate arrays containing wells with diameters of 10, 20, 40, 80, 160, and 1000 μm. The second layout contained four arrays of wells with diameters of 2, 5, 10, and 20 μm. For fabrication, 4-in. silicon wafers (Silicon Quest) were coated with 1μm thick parylene films in a parylene coater (SCS Labcoter 2, Specialty Coating Systems) using 2,2-paracyclophane (Sigma) as the parylene precursor. Wafers were then spin coated with Shin-Etsu Microprime P20 adhesion promoter (3000 rpm, 45 s), then SPR-220 photoresist (Microchem Corp.) to a 1 μm thickness (3000 rpm, 45 s). Wafers were soft baked (115°C, 45 s), exposed for 8 s using a contact mask aligner, hard baked (115°C, 45 s), developed in CD-26 developer (1 min), rinsed in H_2_O and dried with N_2_. Exposed regions of parylene were then etched with O_2_ plasma in an Oxford Plasmalab 100 reactive ion etching system at a parylene etch rate of 60 nm/min. Protein transfer through etched parylene regions was verified by absorbing BSA over the parylene stencils and then performing the dry lift-off, resulting in the formation of homogenous protein layers 5–6 nm in thickness, roughly twice the expected thickness of a BSA monolayer (S1*A* Fig) [30]. Transfer of bacterial cells through the stencils was also verified using the same procedure (S1*B* Fig). After O_2_ plasma etching, deep reactive ion etching using a Bosch process was used to form microwells in the exposed silicon. A Si etch rate of 1.1 μm per cycle was measured, and 18 cycles of the Bosch etch process were performed to obtain a nominal well depth of 20 μm, as verified with a stylus profilometer. Wells were finally etched with O_2_ plasma for 2 min to remove residual photoresist, rendering the surface rich in SiO_2_ groups. Substrates were diced into 15×15 mm coupons and stored in ambient conditions until use.

### Protein functionalization in microwell arrays

Microwell arrays were functionalized with unlabeled WGA (Triticum vulgare) lectin (Sigma), WGA-A488 (Invitrogen) or BSA (Sigma). WGA coatings were used to promote cell adhesion and viability. Although *E*. *coli* was found to have high association with WGA in solution, WGA well coatings did not significantly alter the distribution of cell populations relative to non-specific (BSA) coatings, but did provide higher cell viability after attachment compared to wells with no coating. (S2 Fig). This was likely due to the fact that the lectin coated surfaces trapped moisture within the wells, causing less dehydration during the dry lift-off step. Other well pre-treatments designed to keep cells hydrated during the lift-off process, such as coating the wells with agar, have shown similar improvements in cell viability [31]. Additionally, lectin-coated wells may bias the capture of highly motile, viable cells through adhesive exopolysaccharride binding interactions. For well functionalization, 100 μL of WGA, WGA-A488, or BSA at 500 μg/mL in 1X PBS was incubated on the microwell substrate for 60 min. in a humidity chamber. Substrates were then washed three times with 1X PBS to remove unbound protein and dried with N_2_.

### Bacterial Culture and Seeding

*E*. *coli* K-12 expressing GFP and mCherry were used as model strains to study bacteria seeding behavior in the microwell array platform. These strains were constructed by chromosomal insertion of the genes encoding GFP or mCherry using a Tn7 site specific transposon system [32]. After construction, these strains were stored in glycerol stocks at -80°C. The fluorescent strains were cultured on LB agar plates (10 g NaCl, 5 g Yeast, 10 g BactoTryptone, 15 g agar per liter) containing 5 μg/mL Gentamicin (37°C, 24 hrs), then stored at 4°C for up to 1 month. For seeding experiments, *E*. *coli* was grown to the logarithmic phase in liquid LB media with 5 μg/mL Gentamicin in a sterile 20 mL glass tube (225 rpm, 37°C), harvested by centrifugation, and re-suspended to the desired concentration in water. Here re-suspension in water was preferable, as re-suspension in media resulted in the deposition of salt crystals that interfered with the microscopy. A 100 μL volume of this solution was pipetted onto the WGA-functionalized microwell arrays and incubated in humid conditions for 1 hr. The solution was then aspirated from the substrate and allowed to dry. The parylene film was then removed using a small piece of Scotch^™^ tape and tweezers (Fig 1*B*). These substrates were then imaged with epifluorescence using FITC or TRITC filter sets. Camera settings were adjusted such that individual *E*. *coli* cells expressing GFP or expressing mCherry gave the same average signal intensity.

*P*. *aeruginosa* PA-01 expressing GFP was used for growth studies. The plasmid attTn7::GFP was used for GFP expression in native PA-01, this strain was constructed in the J. Mougous Laboratory (University of Washington, Seattle, WA) and stored in glycerol stocks at -80°C upon receipt. For solid culture, *P*. *aeruginosa* was grown on LB agar plates (30°C, 24 hrs) then stored at 4°C for up to 1 week. Prior to seeding, this strain was cultured to the log phase in liquid LB (30°C, 225 rpm), harvested and re-suspended to an OD_600_ of 0.25. Here, cells were re-suspended into fresh LB media to maintain cell viability during the seeding process. Cells were then incubated over BSA-coated microwell arrays for 1 hr. Substrates were then briefly rinsed in water for ~10 s, dried, and then the parylene was removed from the surface.

### Growth studies of *P*. *aeruginosa*

Agar-coated coverslips were used to trap bacteria cells while providing aqueous chemical media into the wells. For agar-coating, sterile 25×75 mm glass coverslips (Schott Nexterion) were placed in a 3.5 inch diameter polystryene petri dish setting on a hot plate at 50°C, and 10 mL of boiling liquid LB-agar was poured evenly over the coverslips. Excess LB-agar was then removed with a pipette, and the dish was cooled for 30 min at 4°C to gel the agar onto the coverslip with minimal dehydration. The resulting thickness of the hydrated agar layer was estimated to be approximately one hundred microns in thickness. The coverslip was removed from the dish with a razor blade and contacted with a seeded microwell substrate, then immediately placed in a humidified, live cell incubation chamber (In Vivo Scientific, St. Louis, MO) set on an inverted fluorescence microscope stage at 30°C. Growth was studied under these conditions using four replicate experiments.

### Instrumentation

Scanning Electron Microscopy (SEM): A Carl Ziess Merlin SEM was used to characterize the structural features of the microwell arrays after etching and parylene removal. Images were acquired while operating at 3.0 kV. Samples were imaged with a 30° and 45° tilt to provide images of the well sidewalls.

### Fluorescence Microscopy

A Nikon Eclipse Ti-U inverted microscope (10×, NA 0.3 or 20×, NA 0.4) equipped with an Optiscan motorized XYZ stage was used to image the microwell arrays. Fluorescent images were taken using a DS-QiMc monochrome digital camera, FITC and TRITC filter sets, and NS-Elements software. Prior to growth studies, imaging conditions were optimized such that no detectable photobleaching occurred. A neutral density filter was used to adjust the excitation source to 25% of the standard light intensity. Under this setting, no significant bleaching of the cells was observed when imaging with the 10× objective for repeated 100 ms exposures. Time-lapse images were taken of the arrays during growth with the 10× objective at the specified camera settings (100 ms exposure, 16× gain, 1280×1024 binning). Arrays were imaged once every 30 to 60 minutes, and the resulting image sequences were used to generate growth curves.

### Confocal Microscopy

Three-dimensional images of *P*. *aeruginosa* grown to various levels in 10 μm diameter microwells were taken using a Zeiss LSM 710 laser-scanning confocal with Zen 2010 software. Z-stacks were taken using a 20X objective with a 2.5 μm step. Zen 2010 image analysis software was used to re-construct three-dimensional images of microbial cells in the microwells.

### Image Analysis

Grey-scale images of fluorescent well intensities were quantitatively analyzed using ImageJ software. A microarray analysis plugin (http://rsb.info.nih.gov) was used to quantify fluorescent intensity levels of bacterial cells isolated in wells. Here, each well was treated as an independent region of interest, and fluorescent intensity was averaged across the entire area of the well. Standard deviations were then calculated from average signals across replicate wells. Due to uneven background fluorescence from the agar coating, local background levels were also taken immediately next to individual wells. The local background was then subtracted from the average fluorescence intensity of the well. For the seeding and growth studies involving *E*.*coli* expressing GFP, fluorescent signal intensity was also measured across a population of cells, allowing for the determination of average fluorescent signal per cell. This allowed for estimation of the number of cells per well from average well fluorescent signal intensities. For growth studies involving *P*. *aeruginosa* expressing GFP, cell volumes were determined from confocal microscope Z-stack images exported from Zen 2010 software using a volume reconstruction plugin and then correlated to the fluorescent signals measured on the epi-fluorescent microscope system (S3 Fig).

### Statistical Analysis of Data

All data is reported as the average ± standard deviation. The variation in cell seeding levels was described by *CV*_well_:

$$\text{C}{\text{V}}_{\text{well}}\;=\;\frac{{\text{StDev}}_{\text{D}}\;\left(\text{A}.\text{U}.\right)}{\text{Fl}{\;}_{\text{Ave},\text{D}}\left(\text{A}.\text{U}.\right)}$$ (1)

where Fl_Ave,D_ is the averaged fluorescent well intensity after local background subtraction across replicate wells of diameter D, and StDev_D_ is the corresponding standard deviation of replicate wells within an array.

## Results and Discussion

### Characterization of the Physicochemical Microwell Environment

**Fig 2:**
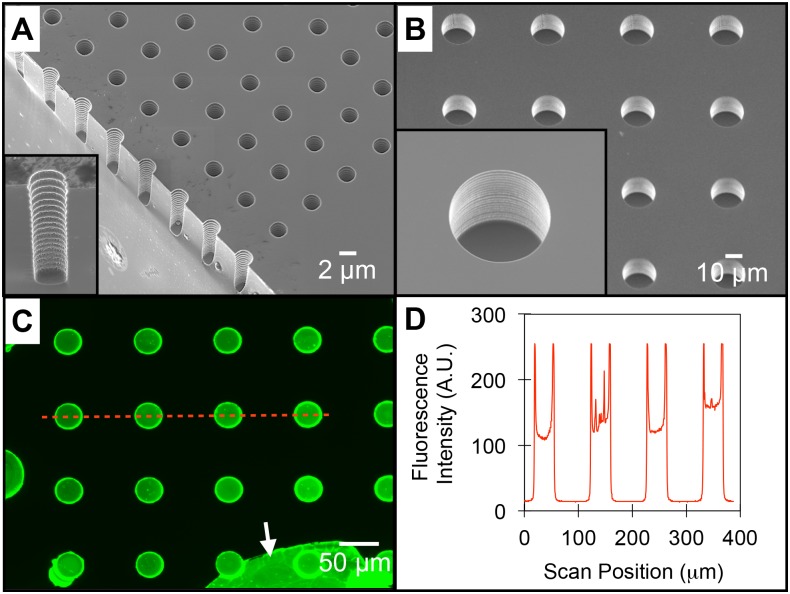
Characterization of the physicochemical features of microwell arrays. (A) SEM image of an array of 2μm diameter microwells and of an individual well after substrate cleavage (inset). (B) SEM images of a 20μm diameter microwell array. (C) Fluorescent image of microwells after functionalization with WGA-A488 and dry lift-off. The arrow highlights a portion of un-peeled parylene, also containing adsorbed WGA-A488. (D) Fluorescent line plot of WGA-A488 coated wells corresponding to the red line in 2C.

The microwell fabrication process was designed to allow for systematic control of both the physical structures and chemical surface features of the well interface. The well features were physically and chemically characterized prior to bacterial seeding. SEM images of low and high-aspect ratio wells demonstrate the capability to tune the level of spatial confinement into which the bacterial communities are assembled (Fig 2*A* and 2*B*). Wells contain walls with a periodic ribbed structure and a smooth floor, characteristic of the Bosch etching process (Fig 2*A* *insert*). Well surfaces can also be modified with desired organic or biological components using liquid or vapor deposition over the entire stencil (Fig 1*A*, step iv). During this functionalization step, the silicon well surface is rich in Si-OH groups, making it amenable to direct contact with aqueous-phase solutions and reactive to organic silane-reagents. To demonstrate modification of the well surface, a solution of fluorescently labeled, adhesive lectin protein (wheat germ agglutinin-AlexaFluor 488, WGA-A488) was incubated over the entire substrate. After washing and removal of the parylene mask, fluorescent images show the protein exclusively coated within the well surface, with higher intensity levels noted at the well edges due to the three-dimensional wall structure (Fig 2*C* and 2*D*). The variation in protein density between wells within an array was 16%. This level of variation is likely due to protein aggregation that occurs across the well surface, and is comparable to the variation previously noted using microcontact printing methods (11–13%) [33]. Further improvements in protein uniformity can likely be obtained using printing buffers that minimize aggregation [27]. Background regions appear unmodified, which was expected as proteins do not diffuse through the parylene layer.

### Seeding Behavior of Bacterial Cells Into the Microwell Array

**Fig 3:**
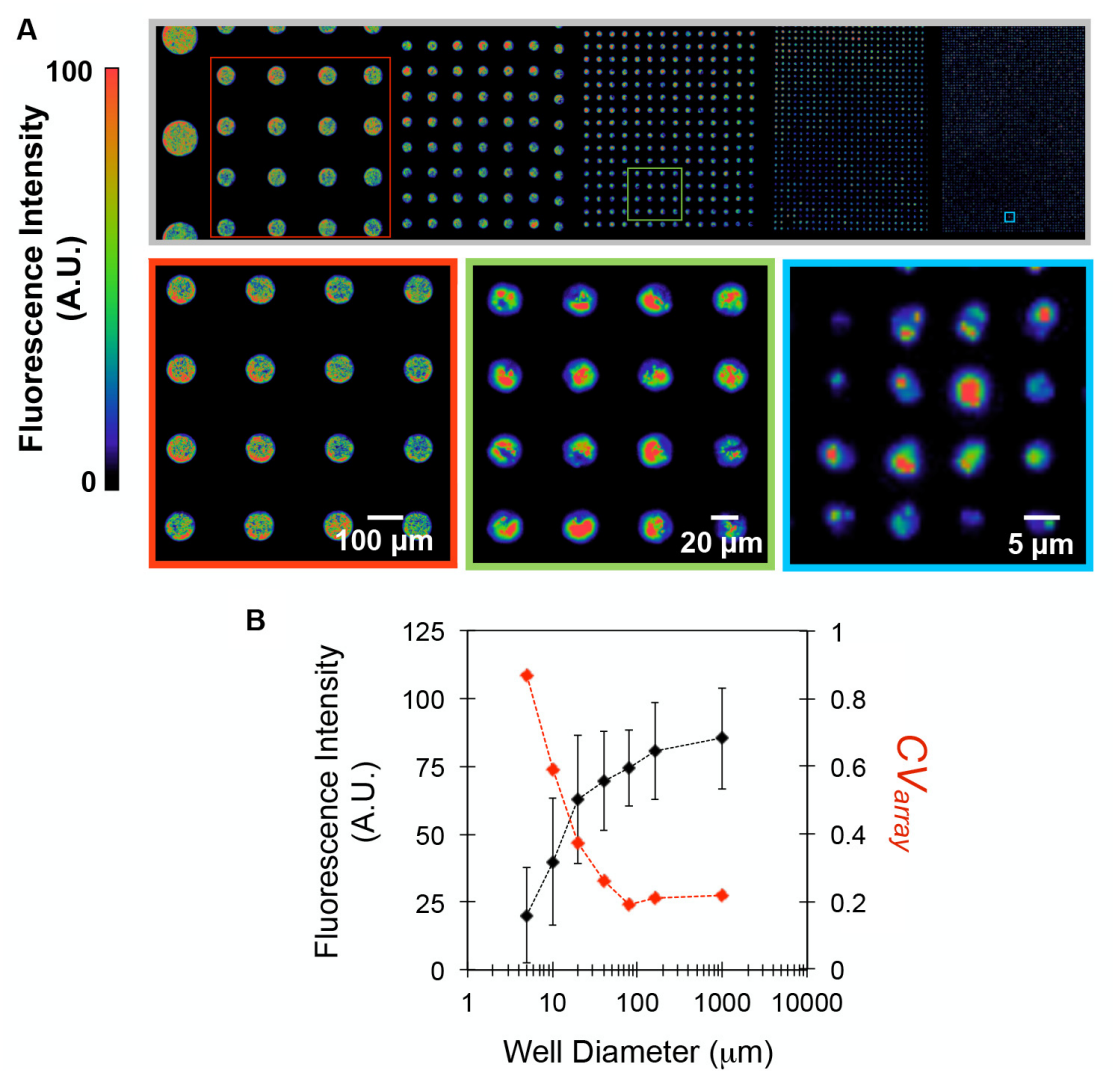
The distribution of bacteria seeded in microwell arrays is guided by well diameter. (A) Mosaic 10X false-color fluorescent image of a combinatorial microwell array after seeding *E*. *coli*-GFP at OD_600_ = 0.3 and dry lift-off to remove background cells. The false color scale denotes fluorescent signal intensities indicative of cell densities. (B) Averaged well fluorescence intensities ± standard deviation measured from individual wells within each array (black line) and *CV*_array_ (red dashed line), the standard deviation divided by the average fluorescent signal for each well diameter.

Control of the physical and chemical features of the microwell interface can be used to bias the bacterial populations isolated during the seeding step. It is well known that the attachment of bacterial cells to a solid interface depends on topological and chemical surface features, and numerous reports have used micro and nanofabrication strategies to control these properties, either to promote or inhibit bacteria attachment and interactions [34–36]. Here, substrates containing arrays of wells with diameters ranging from 5 to 1000 μm were used to investigate the effect of diameter on the population distribution of seeded *Escherichia coli* cells expressing green fluorescent protein (GFP). False color fluorescent images generated from cells isolated in wells after seeding and lift-off reflect the cell densities in the wells (Fig 3*A*). As evident, cells are seeded exclusively within the well boundaries. Magnified images of representative 4×4 arrays qualitatively demonstrate that arrays of smaller wells contain increasingly heterogeneous numbers of cells within initial populations.

**Fig 4:**
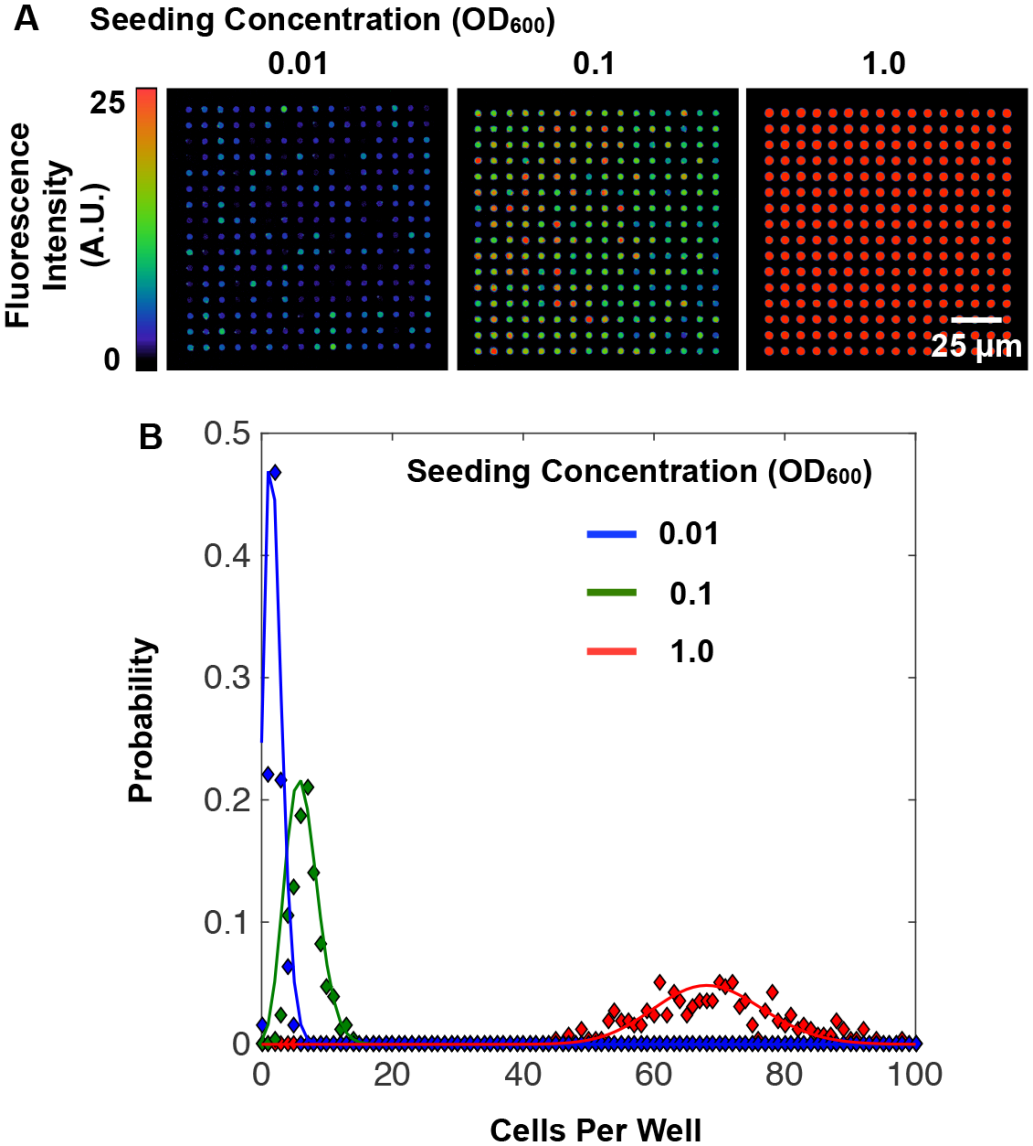
Bacterial well populations follow a Poisson distribution. (A) 20X false color fluorescent images of 5 μm diameter wells seeded with *E*.*coli*-GFP at OD_600_ = 0.01, 0.1, and 1.0. (B) Probability distributions for cell populations at the varied seeding concentrations. Diamonds represent data and solid lines represent a Poisson distribution fit to the data according to eq 2. Seeding at an OD_600_ of 0.01, 0.1, and 1.0 resulted in a ***λ*** value of 1.9, 6.2, and 68.6, respectively, and an ***A*** value of 1.65, 1.35, and 1.00, respectively.

To quantify the trends shown in Fig 3A, the average fluorescence intensity and the variation in signal between replicate wells within the same array (*CV*_array_) were measured for each diameter (Fig 3*B*). Here, average well intensity reflects the average number of cells contained within an array, while *CV*_array_ reflects the variation in initial cell populations present in wells across an array. Populations assembled within larger wells (≥80 μm diameter) allowed for reproducible cell populations (*CV*_array_ ≤ 0.2) to distribute in the well, likely due to minimal crowding or interference from the sidewalls. As well diameters decreased below 80 μm, the average fluorescent signal also decreased, despite larger surface-area to volume ratios for cell attachment. This can be attributed to higher levels of spatial confinement, rendering a higher fraction of well binding sites inaccessible to cells. Also, stochastic interactions between single cells and wells become more pronounced at smaller diameters, causing larger *CV*_array_ values. Similar trends have been noted while seeding mammalian cells into microscale wells [37], and also while encapsulating molecular systems into nanoscale compartments, such as DNA probe functionalization in nanowells for digital PCR systems [38]. To further characterize the population distribution in seeded wells, *E*. *coli*-GFP was seeded into 5 μm diameter wells over a two order of magnitude concentration range (OD_600_ = 0.01 to 1.0, Fig 4*A*). The corresponding frequency histograms (Fig 4*B*) indicate that initial well populations follow a Poisson distribution, suggesting that seeding occurs as independent, random events with frequencies proportional to the concentration of cells present in bulk solution. To mathematically describe the relation between seeding concentration and cell distribution within these wells, the data was fit according to the probability distribution function:

$$P\left(x\right)=A\cdot \frac{\lambda^{x}}{x!}\cdot e^{-\lambda };\;x=0,1,\ldots ,n$$ (2)

where ***x*** represents the number of occurrences (cells captured per well), ***A*** is an amplitude parameter, ***λ*** represents the mean value, ***n*** represents the total number of cells present, and ***P***(***x***) represents the probability of capturing ***x*** number of cells in a well.

These findings suggest that well dimensions can be used in combination with cell seeding concentration to tune the distribution of initial microbial populations isolated within an array. Control of this distribution is attractive, because it enables parallel monitoring of either a homogenous or heterogeneous assortment of initial populations; both are situations that are informative for characterizing microbial community development. Further optimization of the seeding protocol will allow for improved control of well populations. Currently, spatial correlations can often be identified when inspecting arrays spanning larger areas (~2 mm^2^), likely caused by drying artifacts that occurred on the surface before the lift-off step. However, analysis of a large number of wells minimizes this effect. Finally, while the characterizations were made here with a model *E*. *coli*–GFP system, seeding other species may result in changes to population distributions, driven by differences in microbial traits (e.g. motility, extracellular matrix composition, cell-surface and cell-cell affinities). However, it can be expected that similar transitions to highly heterogeneous population assembly will occur for any species as the size of the well approaches the scale of individual cells. Moreover, quantifying deviations from Poisson-like distributions of microbes that result during the seeding process may be used to screen for microbe biases or affinities for, or against, particular surfaces or other microbes.

### Trapping and Growth of *P*. *aeruginosa* in Microwell Arrays

After seeding, the chemical environment within the wells was controlled by sealing the microwell substrate with an agar-coated coverslip that had been treated with the desired chemical media (Fig 1*A*, step vii). This provided a physical barrier to trap motile cells within the well structures. The successful trapping of motile bacterial populations was demonstrated using *P*. *aeruginosa* modified to express GFP, which showed both surface-attached and un-attached populations confined within the wells during real-time monitoring (S1 Video). The motility observed from un-attached cells suggested that a large fraction of cells remained viable through the seeding and trapping process. Similar observations were made while monitoring bacterial cell populations confined in smaller well volumes (S2 Video).

After trapping, a small number of cells were also found in background regions, which was caused by cell removal from wells during contact of the seeded substrate with the agar coverslip. Background cells appeared to be trapped between the agar and the silicon interface and typically showed no motility over time. While these cells also have the potential for growth [39], they are few relative to the number that remain trapped within the well volume and usually cause no interference with the test sites. Due to the static nature of media in the wells the after trapping step, it is expected that mass transfer across the arrays will be diffusion-limited. However, uniform concentration of oxygen and nutrients across the arrays is expected due to the small array area (200 x 200 μm). For example, the diffusivity of oxygen in a 1.5% (w/v) agar gel at 30°C is D_O2,agar_ = 2.70 x 10^−5^ cm^2^/s [40], and the characteristic time-scale for diffusion across the length of this array is ~10 s, well below the time scale required for growth (hours).

After well sealing with agar-coated coverslips, *P*. *aeruginosa* was monitored for growth in wells with diameters between 5 and 20 μm, where the initial population distribution (*CV*_well_) was shown to be highly dependent on well diameter (Fig 3B). Here, Luria-Bertani (LB) media was added in the agar coverslip coating to promote growth. Growth was observed by monitoring well arrays with time-lapse fluorescence microscopy and then quantified in terms of cell volume fraction, the measured cell volume normalized to the overall volume of the well. This metric was determined using a correlation curve relating the average well fluorescence intensity (A.U.), obtained from the epi-fluorescence microscope system, to cell volume, quantified with a confocal microscope (S3 Fig).

**Fig 5:**
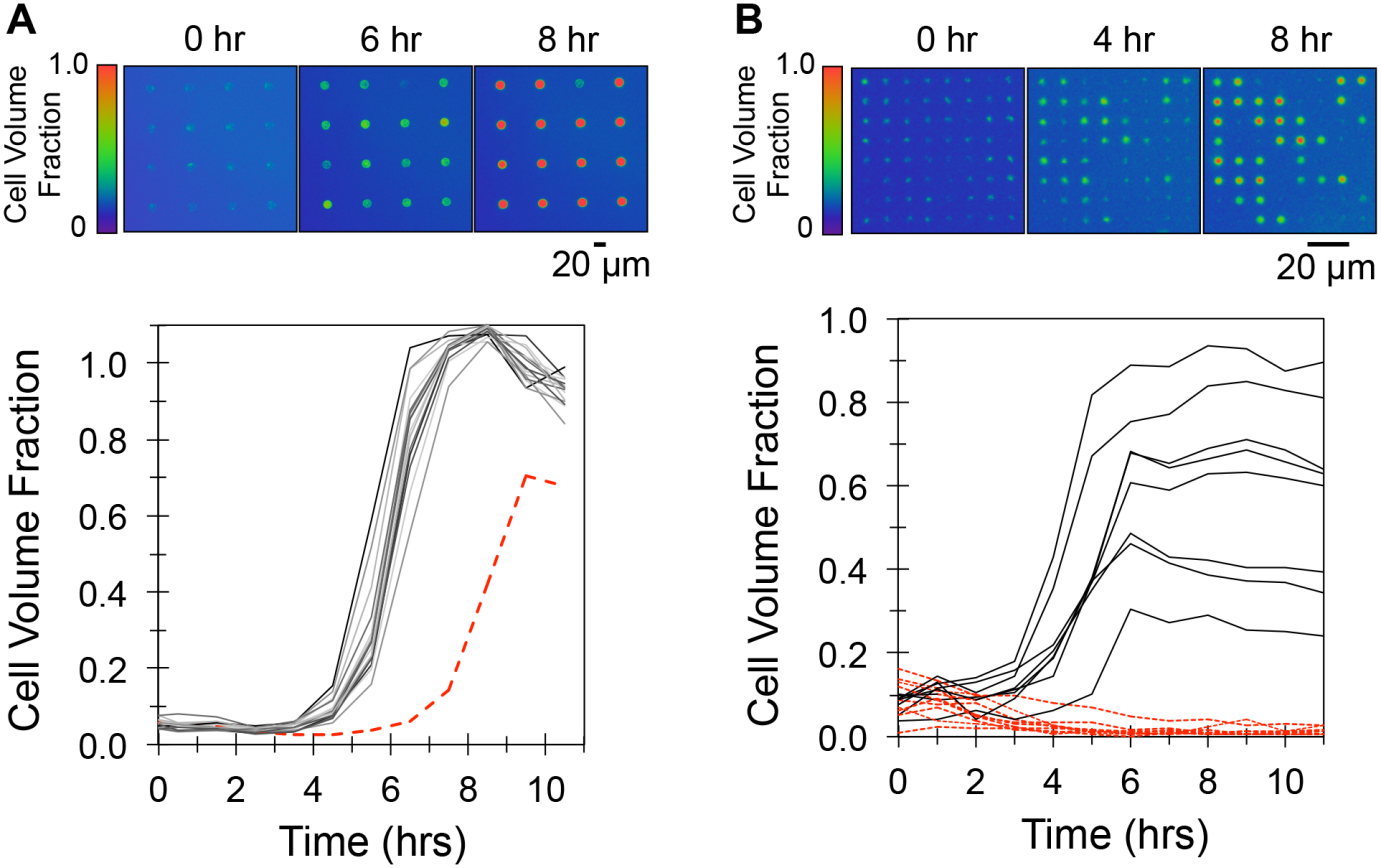
*P*. *aeruginosa* growth trajectories in 5 and 20 μm diameter microwell arrays. (A) Top: False-color fluorescent images of growth in 20 μm diameter arrays. Bottom: Corresponding growth trajectories. The dashed red trajectory indicates growth in an outlier well. (B) Top: False-color fluorescent images of growth in 5 μm diameter arrays. Solid black trajectories denote wells where growth and colonization occurred, dashed red trajectories denote wells where decay and extinction occurred. Data is representative of 4 independent growth experiments.

At the initial time point, broader distributions of *P*. *aeruginosa* populations were noted in wells with smaller diameters (*CV*_5μm well_ = 0.68 ± 0.08; *CV*_20μm well_ = 0.28 ± 0.04, n = 4), consistent with the trends noted previously (Fig 3B). The fluorescent signals measured from wells during incubation indicated that growth occurred over a 10 hr period. Cells in 20 μm diameter wells had a 4 hr lag phase followed by 4 hr growth period until the cell volume reached the final volume of the well (Fig 5*A*, S3 Video). A small over-estimation in cell volume, indicated by cell volume fractions slightly higher than 1.0, was noted at late growth times, and was likely caused by increases in cell density as cells fill the entire well volume. Similar effects have been noted while monitoring *P*. *aeruginosa* growth in other confined systems [14]. After growth, fluorescence intensity remained stable for at least 40 hr. Upon inspection of these wells under fluorescence, bright cellular aggregates appeared to be present, but no cellular motility could be detected within the cell mass. An example of 20 μm diameter wells after growth is shown in real-time playback (S4 Video). The lack of cellular motility here is in stark contrast to that seen immediately after seeding (S1 and S2 Videos), where high cellular-motility is easily observed. This suggests the possible establishment of biofilms within the wells during the growth period. Also apparent is the presence of an outlier well, which occurred in the 20 μm diameter arrays with a frequency of 5–10% (n = 4). However, the relatively low variation in initial cell populations assembled under these seeding conditions allowed for directed, repeatable growth trajectories, which enables averages of trajectories to be determined. This demonstrates the utility of this platform as a ‘high-statistics’ method for monitoring growth kinetics of replicate, microscale bacterial populations in controlled microenvironments. Cells in 5 and 10 μm diameter microwell arrays showed strikingly different growth behavior. In 5 μm diameter wells, a wide variety of trajectories were measured (Fig 5*B*, S5 Video), ranging from an increase in signal intensity due to growth (< 4 hr to stationary phase), to population decay and extinction, indicated by a decaying fluorescence signal. The decay in fluorescent signal is due to cell lysis and extracellular GFP diffusion, which was previously shown using 60X time-lapse fluorescent microscopy to monitor individual cells during lysis [41]. Similar behavior was also noted in 10 μm diameter wells, and replicate experiments consistently showed this behavior to persist within smaller (5, 10 μm diameter) wells, whereas 20 μm diameter wells provided reproducible growth outcomes (S4 Fig).

**Fig 6:**
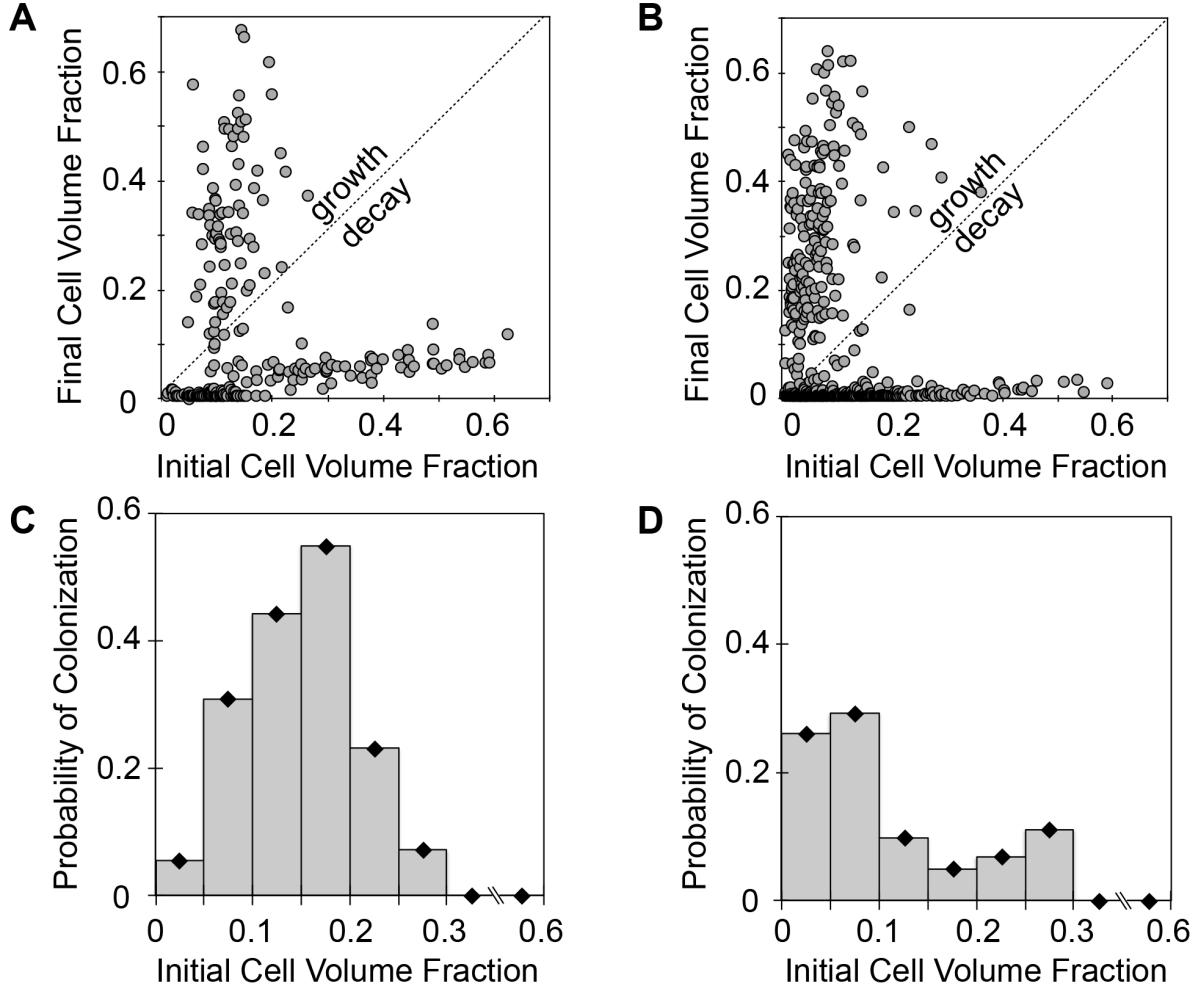
Growth of *P*. *aeruginosa* in confined volumes depends on inoculum levels. (A) Scatter plots of initial and final (t = 24 hrs) cell volume fraction in 10 μm diameter wells and (B) 5 μm diameter wells. Growth-decay line deciphers wells that increased or decreased in cell numbers over the incubation period. (C) Probability of well colonization with initial volume fraction of seeded cells for in 10 μm diameter and (D) 5 μm diameter wells. Data was taken from n = 256 wells for 10 μm diameter arrays and n = 840 wells for 5 μm diameter arrays from 4 independent growth experiments.

We hypothesized that the variation in growth response in the 5 and 10 μm diameter wells could be attributed to the broader distributions of initial populations seeded into the wells. To investigate this further, we compared the initial and final (t = 24 hrs incubation) cell volumes from 5 and 10 μm wells (n = 4 experiments), generating hundreds of independent growth trials (Fig 6*A* and 6*B*). Additionally, the probability of well colonization, defined as the percentage of wells showing a significant increase in cell volume after incubation, was computed (Fig 6*C* and 6*D*). As evident, the probability for colonization was highly dependent on initial conditions. In the case of the 10 μm diameter wells, colonization was possible when wells were inoculated up to a volume fraction of 0.3. Within this region, an inoculation range where the probability of colonization was the highest appears at cell volume fractions between 0.1 and 0.15. Comparable trends were found within the 5 μm wells, where the probability of colonization was highest at cell volume fractions between 0.01 and 0.1 and diminished between 0.1 and 0.3. In both cases, colonization did not occur when initial well volumes were greater than 0.3. It is likely that decay occurs at this inoculum level due to over-consumption of resources during the early stages of growth, since nutrient exchange is limited in the confined environments. Although additional analysis is required to test this hypothesis, these findings point towards the importance of spatial confinement in community development. In addition, these results demonstrate the first successful application of this platform to screen hundreds of unique, independent bacterial populations, driven by highly heterogeneous population assembly in small wells, for the discovery of populations and environmental conditions that influence colony growth.

### Assembly of Multi-Component Bacterial Communities

In addition to single-species colonies, dynamic interactions within multi-species populations can be investigated using this platform. Microbial communities are often shaped by cooperative, competitive, or pathogenic interactions between different species, and recently several pair-wise interactions have been shown to be critical in driving community phenotype [42]. However, the vast majority of interactions occurring within poly-microbial communities are unknown, but likely depend on the relative abundance of interacting members present [16]. The high-throughput nature inherent to this method makes it attractive for characterizing inter-species interactions after assembling either homogenous or heterogeneous populations, similar to the single-species systems previously described.

**Fig 7:**
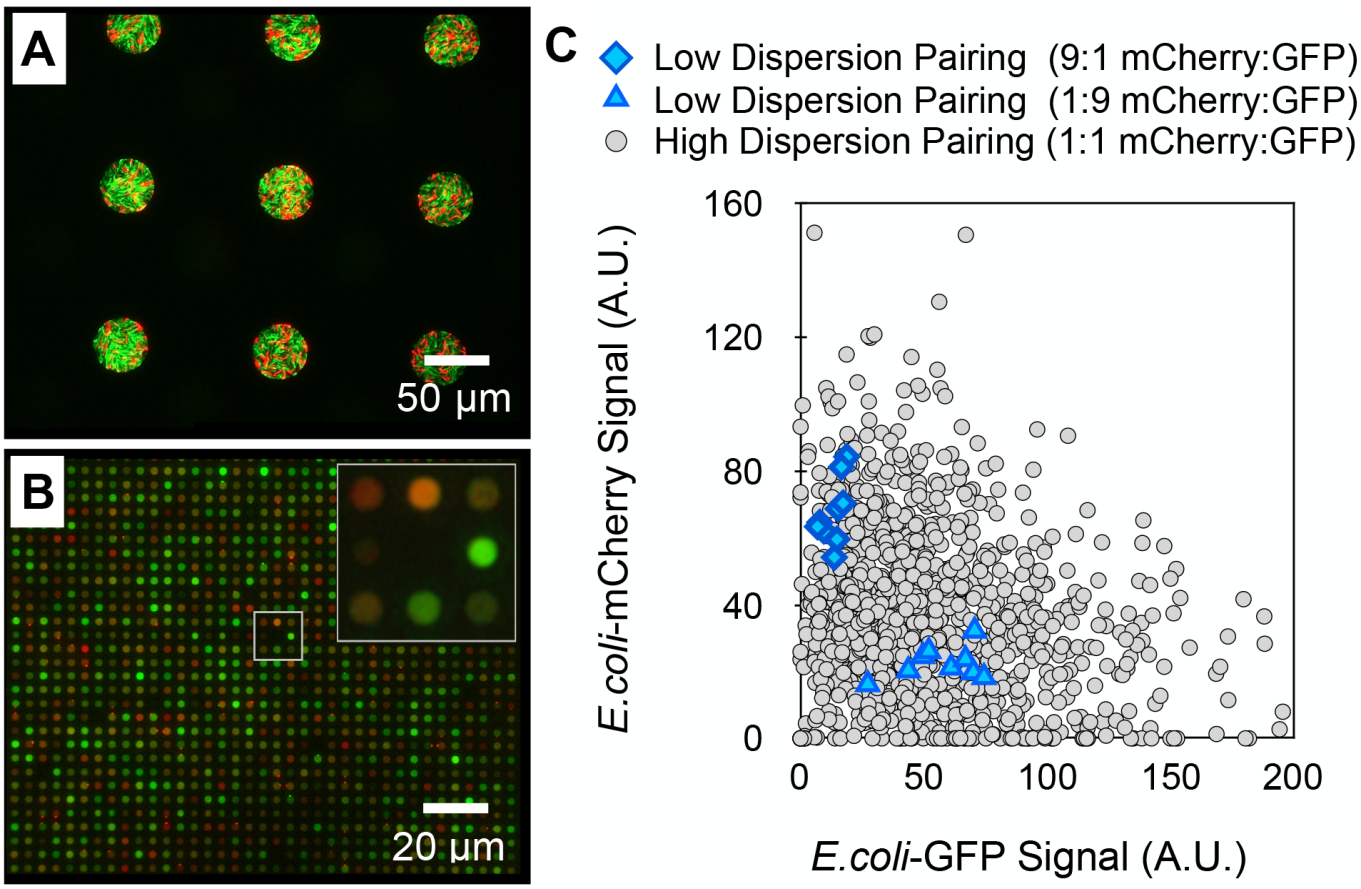
Multi-member bacterial communities can be assembled at low or high dispersion. (A) Low dispersion pairing: Seeding a 1:9 mixture of *E*. *coli*-mCherry (red) and *E*. *coli*-GFP (green) at an overall OD_600_ of 0.4 into 40 μm diameter microwell arrays. (B) High dispersion pairing: Seeding a 1:1 mixture of *E*. *coli*-mCherry and *E*. *coli*-GFP into 2 μm diameter arrays at an overall OD_600_ of 1.0. (C) Scatter plot of GFP and mCherry signals after low or high dispersion pairing.

Here, the assembly of a model, two-component *E*. *coli* system constitutively expressing mCherry or GFP into large (40 μm diameter) wells promoting homogenous population assembly, or small (2 μm diameter) wells promoting heterogeneous population assembly was examined. In large wells, assembled *E*. *coli*-mCherry and GFP populations had a reproducible mCherry (red) to GFP (green) signal ratio of 0.39 ± 0.09 (Fig 7*A*), reflective of the ratio at which the pair was mixed together in solution, demonstrating pairing at low dispersity. In stark contrast, these cells were paired with high dispersity in small wells, providing a highly heterogeneous distribution of initial populations, as noted by a variety of unique GFP and mCherry signatures (Fig 7*B*). A scatter plot of GFP-mCherry signal intensities generated from individual wells in each case contrasts the differences between initial population dispersions (Fig 7*C*). This finding demonstrates the potential use of this platform to measure growth and interactions between replicate, multi-member populations, or to screen unique combinations of interacting pairs in a manner similar to the single-component systems previously described. This unlocks a new, transformative approach for studying fitness, competition, mutualism, or pathogenicity across a vast parameter space using a single substrate.

## Conclusions

A high-throughput platform for measuring the growth of independent microbial populations in three-dimensional, microscale landscapes with controlled physical and chemical features facilitates exploring the complex parameter space that influences microbial community development. Central to this methodology is the ability to isolate microbial cells precisely into wells using a parylene-based lift-off technique, combined with the capability of assembling initial populations with tunable dispersity by controlling the geometric features of the wells. We have demonstrated that seeded populations of bacteria can be trapped in three-dimensional microwells under appropriate environmental conditions, allowing for dynamic growth or decay measurements across a large number of independent populations using a simple, quantitative fluorescence readout.

The ability to tune the initial population dispersity using microwell diameter and depth is attractive because it allows for the study of community behavior under different environmental and initial conditions. Seeding in wells with diameters significantly greater than the size of individual cells drives homogenous population assembly (*CV*_well_ ≤ 0.20), enabling a ‘high-statistics’ approach to monitoring the growth or decay of replicate populations. Conversely, seeding into wells with diameters that approach the size scale of individual bacterium drives heterogeneous population assembly, enabling one to screen a large parameter space in order to identify cellular combinations and environments that are conducive or inhibitory to community growth and proliferation under prescribed conditions. Future work is aimed at screening interactions using high-dispersion population assembly with multi-species microbial communities in order to uncover symbiotic, mutualistic, and pathogenic relationships.

## Supporting Information

S1 Fig AFM characterization of protein and bacterial cell patterning on silicon substrates using the parylene dry lift-off process. (A) AFM image of BSA patterned into 20 μm diameter spots and (B) AFM image of *E*.*coli* cells patterned as 5 μm wide parallel lines.
(TIF)

S2 Fig Characterization of *E*.*coli* seeding using WGA-coated microwells. (A) 20X fluorescent-brightfield images of native *E*.*coli* after staining with WGA-A488 in the absence (top) or presence (bottom) of the complementary oligosaccharide (50 mM GlcNAc), verifying the binding specificity of WGA to GlcNAc expressed in the extracellular matrix of *E*.*coli*. (B) Comparison of population distributions of *E*.*coli* expressing GFP after seeding into 5 μm diameter microwells coated with WGA or BSA. (C) Fraction of dead cells within wells after staining with a live/dead assay.
(TIF)

S3 Fig Correlation between well fluorescent signals measured with the epi-fluorescent microscope system under standard settings (10X objective, 100 ms exposure, 16× gain, 1280×1024 binning) and cell volume measurements taken with a confocal microscope for *P*. *aeruginosa* expressing GFP. (A) False-color fluorescent images from the epi-fluorescent system (top row), and corresponding confocal microscope images (middle and bottom row) after cell growth to different levels in 10 μm diameter wells. Dashed white lines denote well boundaries. (B) Resulting correlation curve relating fluorescent intensity values (A.U.) to cell volume fraction. Cell volume fraction was taken to be the total volume of cells within a well divided by the overall volume of the well.
(TIF)

S4 Fig False color fluorescent images of *P*. *aeruginosa* growth after seeding. (Initial) and incubation (final, t = 24 hrs) at 30°C in arrays containing wells of diameters 5, 10, and 20 μm.
(TIF)

S1 Video *P*. *aeruginosa* populations trapped in 160 μm and 80 μm diameter microwells. Populations were seeded in wells then trapped with an agar-coated coverslip and monitored with epifluorescence using a 10X objective. The movie is shown in real-time playback.
(MOV)

S2 Video *P*. *aeruginosa* populations trapped in an array of 40 μm diameter microwells after seeding and trapping with an agar-coated coverslip. The movie was recorded at 10X with epifluorescence and is shown in real-time playback.
(MOV)

S3 Video False-color fluorescent time-lapse image showing *P*. *aeruginosa* colony growth during incubation in 20 μm diameter microwells at 30°C. The movie was recorded over 10 hours with a 1 hr time interval.
(MOV)

S4 Video False-color fluorescent image showing *P*. *aeruginosa* colonies in 20 m diameter wells after 24 hrs of growth. The movie is shown in real-time playback and was recorded under the same camera settings as in S3 Video but with reduced gain to avoid saturation of the fluorescent signal in the wells due to the higher cell densities.
(AVI)

S5 Video False-color fluorescent time-lapse image showing growth or decay of *P*. *aeruginosa* colonies in 5 μm diameter microwells during incubation at 30°C. The movie was recorded using the same settings as S3 Video.
(MOV)
