# Correction: Stochastic Assembly of Bacteria in Microwell Arrays Reveals the Importance of Confinement in Community Development

**DOI:** 10.1371/journal.pone.0160135

**Published:** 2016-07-21

**Authors:** Ryan R. Hansen, Andrea C. Timm, Collin M. Timm, Amber N. Bible, Jennifer L. Morrell-Falvey, Dale A. Pelletier, Michael L. Simpson, Mitchel J. Doktycz, Scott T. Retterer

The middle initial of the first author’s name is incorrect. The correct name is: Ryan R. Hansen. The correct citation is: Hansen RR, Timm AC, Timm CM, Bible AN, Morrell-Falvey JL, Pelletier DA, et al. (2016) Stochastic Assembly of Bacteria in Microwell Arrays Reveals the Importance of Confinement in Community Development. PLoS ONE 11(5): e0155080. doi:10.1371/journal.pone.0155080
