# Supplementary figures and images for: Stochastic Assembly of Bacteria in Microwell Arrays Reveals the Importance of Confinement in Community Development
Source: PLoS One. 2016 May 6;11(5):e0155080. doi: 10.1371/journal.pone.0155080 (PMC4859483; doi:10.1371/journal.pone.0155080)

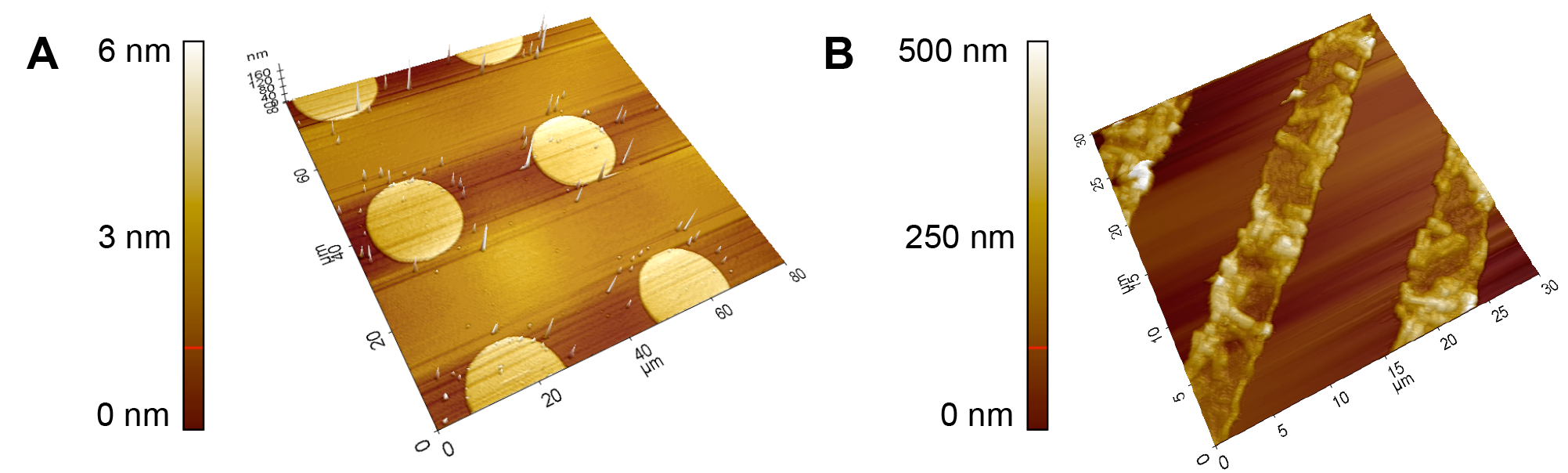

Supplement: S1 Fig — (A) AFM image of BSA patterned into 20 μm diameter spots and (B) AFM image of E.coli cells patterned as 5 μm wide parallel lines. (TIF) [file pone.0155080.s001.tif]

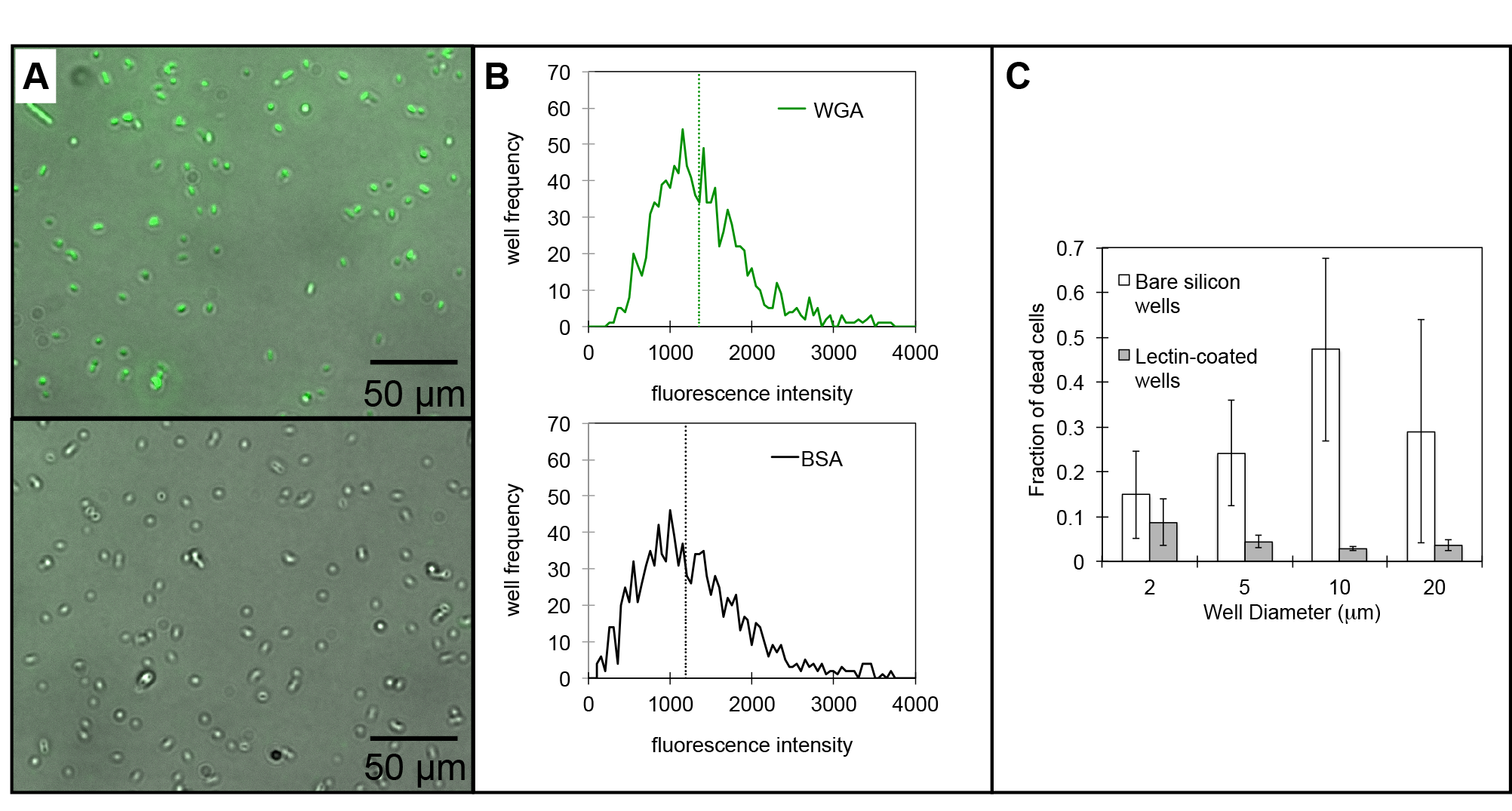

Supplement: S2 Fig — (A) 20X fluorescent-brightfield images of native E.coli after staining with WGA-A488 in the absence (top) or presence (bottom) of the complementary oligosaccharide (50 mM GlcNAc), verifying the binding specificity of WGA to GlcNAc expressed in the extracellular matrix of E.coli. (B) Comparison of population distributions of E.coli expressing GFP after seeding into 5 μm diameter microwells coated with WGA or BSA. (C) Fraction of dead cells within wells after staining with a live/dead assay. (TIF) [file pone.0155080.s002.tif]

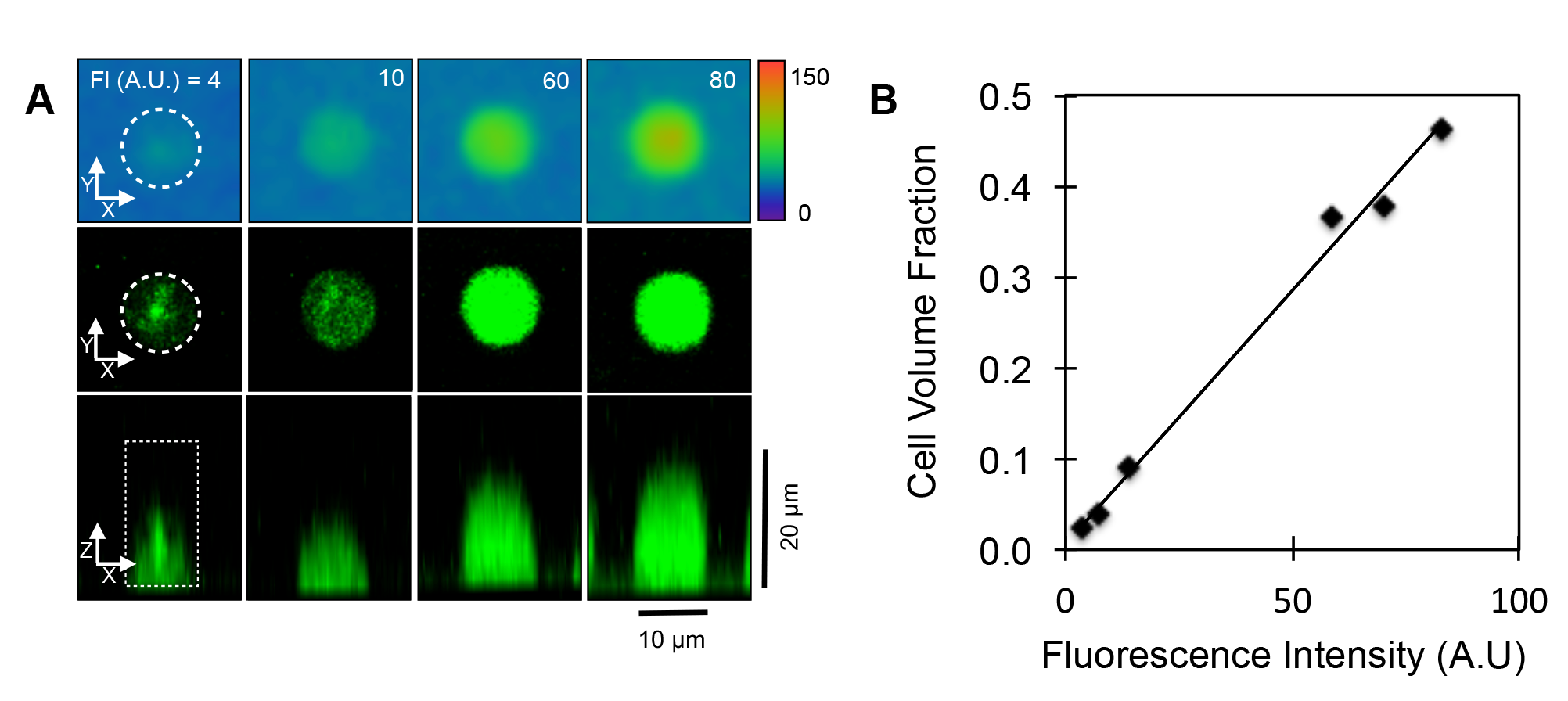

Supplement: S3 Fig — (A) False-color fluorescent images from the epi-fluorescent system (top row), and corresponding confocal microscope images (middle and bottom row) after cell growth to different levels in 10 μm diameter wells. Dashed white lines denote well boundaries. (B) Resulting correlation curve relating fluorescent intensity values (A.U.) to cell volume fraction. Cell volume fraction was taken to be the total volume of cells within a well divided by the overall volume of the well. (TIF) [file pone.0155080.s003.tif]

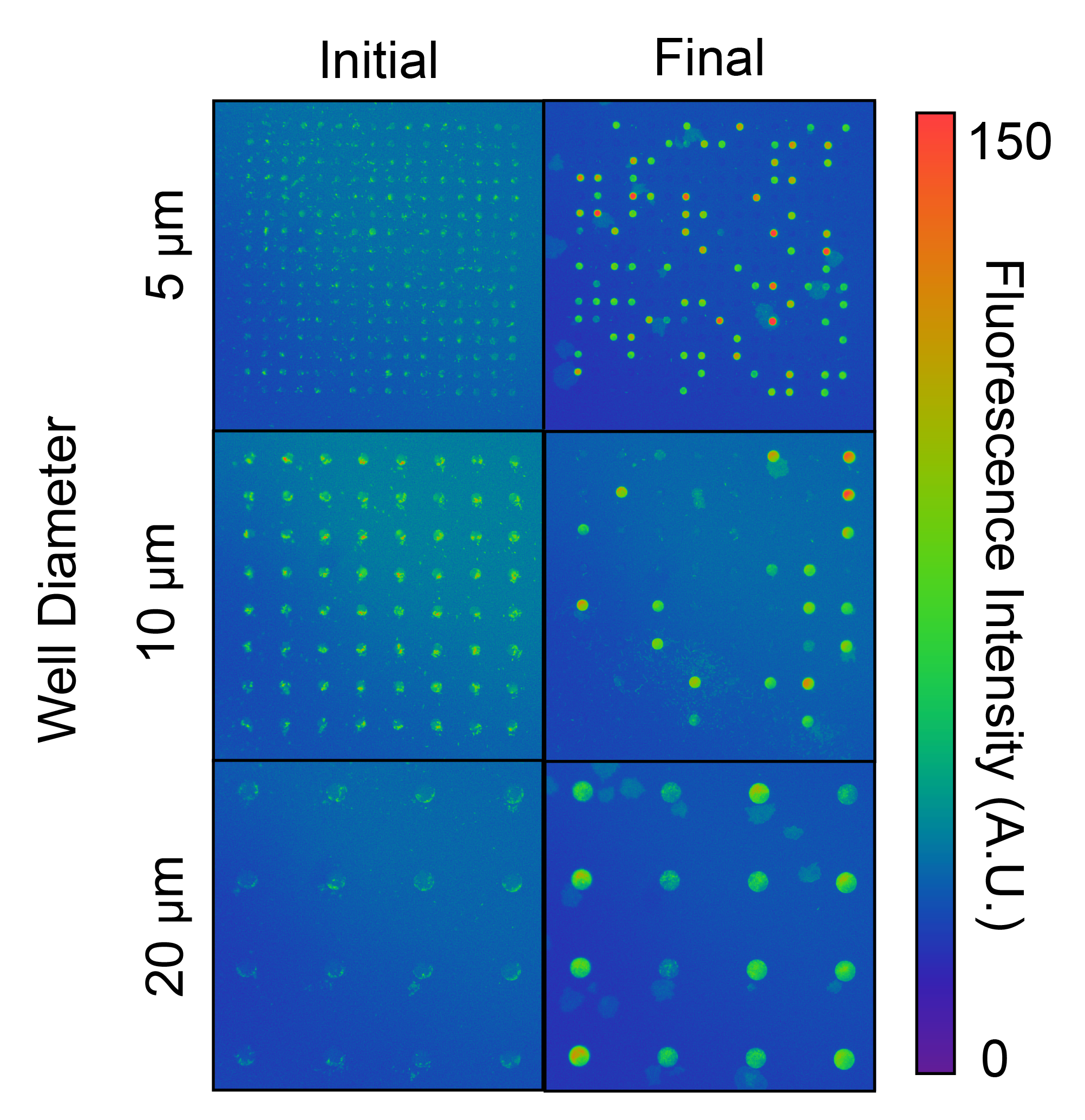

Supplement: S4 Fig — (Initial) and incubation (final, t = 24 hrs) at 30°C in arrays containing wells of diameters 5, 10, and 20 μm. (TIF) [file pone.0155080.s004.tif]
